# Same-day antiretroviral treatment (ART) initiation and associated factors among HIV positive people in Northwest Ethiopia: baseline characteristics of prospective cohort

**DOI:** 10.1186/s13690-020-00473-4

**Published:** 2020-09-22

**Authors:** Nurilign Abebe Moges, Olubukola Adeponle Adesina, Micheal A. Okunlola, Yemane Berhane

**Affiliations:** 1 Department of Public Health, College of Health Sciences, Debre Markos University, Debre Markos, Ethiopia; 2 Pan African University, Life and Earth Sciences Including Health and Agriculture Institute (PAULESI), University of Ibadan, Ibadan, Nigeria; 3 Department of Obstetrics and Gynecology, College of medicine, University of Ibadan, Ibadan, Nigeria; 4 Department of Obstetrics and Gynecology, University College Hospital, Ibadan, Nigeria; 5 Department of Epidemiology, Addis Continental Institute of Public Health, Addis Ababa, Ethiopia

**Keywords:** New HIV patients, Same-day treatment initiation, ART, Ethiopia

## Abstract

**Background:**

Despite a well-established fact that same-day or rapid ART initiation after a positive HIV test result is vital for faster viral suppression and for prevention of further sexual transmissions of HIV, there is a paucity of evidence on the uptake of same-day ART initiation among newly HIV diagnosed people in Northwest, Ethiopia.

**Methods:**

A cross-sectional study was conducted between December 1st, 2018 and July 30, 2019. About 759 newly HIV diagnosed adults were recruited from 24 health facilities. Data were collected using interviewer-administered questionnaire. Data were entered using EPI-Data and exported to SPSS and STATA software for further analysis. Bivariate logistic regression was used to select candidate variables at *p*-value less than 0.25 for multivariate logistic regression. Then adjusted odds ratio with 95% Confidence Interval (CI) at p-value of less than 0.05 was used to declare the statistical associations between the dependent and independent variables.

**Result:**

Magnitude of same-day ART initiation was 318 (41.90%) **[(**95% CI, 38.2–45.20%)]. Factors associated with same-day ART initiation were: Patients resided in West Gojjam Zone were 2.04 times more likely to initiate same-day ART compared to those in Bahir Dar city administration [AOR = 2.04 (1.04–3.97)], patients in the health centers were 3.06 times more likely to initiate same-day ART initiation compared to those in the hospitals [AOR = 3.06 (1.90–4.92)] and Patients who were diagnosed their HIV status at the same health facility where they linked for ART were 2.16 times more likely to initiate ART at the same-day of diagnosis [AOR = 2.16 (91.24–3.74)]. Moreover, patients with no opportunistic infection [AOR = 2.08 (1.04–4.19)] and pregnant women [AOR = 3.97 (1.78–8.87)] were more likely to initiate ART same-day of diagnosis.

**Conclusions:**

Same-day ART initiation was low among HIV patients in Ethiopia. Patients attending their treatment at hospitals and those from big city (Bahir Dar) were less likely to initiate same-day ART. Clinical factors such as having opportunistic infections and non-pregnancy status affected the immediate initiation of treatment. HIV positive people who seek care in hospitals and those tested HIV positive from another health facilities in which they did not intend to continue their ART follow-up care need special attention.

## Background

The World Health Organization (WHO) defines rapid initiation of antiretroviral therapy (ART) as commencement of highly active antiretroviral therapy (HAART) within 7 days of HIV diagnosis. WHO also strongly recommends ART initiation on the same day as HIV diagnosis, after ensuring the person’s willingness and readiness to start ART immediately, unless there are clinical reasons to delay treatment [1]. Ethiopia started implementing the “test and treat’ policy in 2017 [2]. Same-day ART initiation may be a key approach in reaching the 2020 Joint United Nations Programme on HIV/AIDS goal of 90% of all people living with HIV (PLHIV) knowing their status, 90% of those diagnosed receiving sustained ART, and 90% of those receiving ART achieving viral suppression. It may also be important for achieving the suggested fourth “90%” goal: improving health-related quality-of-life in PLHIV [3].

Another benefit of same-day or rapid ART initiation after a positive HIV test result is faster viral suppression and halting further sexual transmission of HIV [4–9]. Rapid ART initiation also decreases HIV related morbidity and mortality [10]. Despite such benefits of rapid ART initiation, delaying the initiation of ART is common in many developing countries [11, 12]. Although there are many benefits of rapid ART initiation, increased LTFU has also been reported in Uganda, Nigeria and South Africa [13–15]. Many reasons have been given for delayed commencement of ART and these include patient’s choice, prolonged adjustment periods, transport costs due to distance from health facility, stigma and fear of disclosure. Other factors associated with delayed ART uptake are staff shortages, long waiting times, fear of drug side effects, male sex, younger age and the need to take time off work [11, 16]. Furthermore, there are factors that have been associated with delay or lack of linkage to care including acquiring HIV through heterosexual contact/injecting drug use, younger age at diagnosis, lower levels of education, feeling well at diagnosis and alternate healing systems [17, 18]. However, the previous studies were undertaken during the pre-test and treat era that emphasized factors associated with poor linkage to ART service, not same-day ART acceptance. In addition, rapid initiation of ART was defined as ranging from 14 to 180 days [10, 11, 19–22]. That may not reflect the rapid treatment acceptance as per the new WHO guideline [1].

In South Africa, the same-day initiation of ART was 40.1% from 2017 to 2018 in which increased from 30.3% in October 2017 to 54.2% in June 2018 [15]. In Taiwan, rapid initiation of ART was 33.8% in 2014 and increasing to 68.3% in 2017 [8]. Similarly in San Diego, 26 and 48% of newly diagnosed HIV positive individuals started ART on the same-day of diagnosis and within a week respectively [23]. Though findings from a systematic review favored same-day initiation of ART, compared with standard care [3] it was challenged from healthcare professionals [5], structural factors including HIV testing site [24, 25] and from patients side [26–28]. In Ethiopia, about 61% of the HIV-positive patients were linked to care immediately after testing positive for HIV [29]. However, it does not mean they were started on ART immediately.

The acceptability of the same-day ART initiation among pregnant and lactating women using prevention of mother to child (PMTCT) option B+ is a well-addressed issue in previous studies [25, 30, 31]. Randomized control trials also demonstrated that the same-day ART initiation is feasible and beneficial for patients [32, 33]. However, findings from these scenario may not be generalizable due to the nature of the study subjects and the environment in which the studies were undertaken.

Although many qualitative studies have been conducted about the barriers and facilitators to same-day ART initiation [12, 34–40], there is a paucity of quantitative evidence in this regard. Hence, the current study aimed to address the previous studies’ methodological gaps by applying advanced quantitative statistical analysis. Furthermore, same-day ART initiation is affected by patient level, facility level, and community level factors [18]. However, such multi-level factors have not been adequately addressed in previous studies because most used secondary data [15, 18].

In Ethiopia, most of the studies that examined factors affecting treatment initiation, late presentation to clinical care, drug adherence and adverse drug reactions used either case-control or retrospective cohort designs [41–46]. These studies used secondary data and were unable to adjust for different confounding factors because of incomplete data from routine services. The current study, therefore, overcomes the limitations of previous studies by incorporating primary data collection in the HIV test and treat strategy. Therefore, the study aimed to determine the magnitude of same-day ART initiation among HIV patients and associated factors in the Ethiopian context.

## Methods and materials

### Study design

This study was part of a prospective cohort study to measure both same-day treatment initiation and major clinical outcomes after 6th and 12th months on ART. Newly diagnosed HIV positive individuals were enrolled to the cohort. The study aimed to determine uptake of same-day ART, estimation of time to antiretroviral treatment initiation, determine predictors of timely initiation of ART and determine treatment outcome that include treatment retention and viral suppression at 6th and 12th months of the follow up. The baseline data of the prospective cohort study was used in the current study to determine the magnitude of same-day ART initiation among HIV patients and associated factors in Ethiopian.

### Study settings

The study was conducted between December 1st, 2018 and July 30, 2019, in Gojjam, Northwest Ethiopia. Gojjam has three administrative zones namely, East Gojjam; West Gojjam and Bahir Dar city administration. The administrative centers/towns are Debre Markos, Finote-Selam and Bahir Dar which are located at 300, 364 and 564-km respectively from the capital city of Addis Ababa. According to the 2007 population census conducted by the Central Statistical Agency of Ethiopia (CSA), East Gojjam zone has a total population of 2,153,937 and West Gojjam Zone including Bahrdar city administration has a total population of 2,106,596 [47].

According to East Gojjam zonal health department report of May 2018, there are 32 ART centers of which 25 are health centers while 7 are hospitals with a total of 16,926 patients. There are 30 ART centers (four hospitals and 26 health centers) in West Gojjam with a total of 9600 patients and similarly, there are 9 ART centers in Bahrdar city administration in which 2 hospitals and 7 health centers with total HIV patients of 5088 December 30, 2019. In all health facilities which are rendering ART service, HIV test and treat program had been started since November 2017. Meanwhile, the Ethiopian national guidelines for comprehensive HIV prevention, care and treatment recommended as a patient should start ART as soon as possible and most should be started in the second visit (after 1 week), while TB patients should initiate ART within two to eight weeks of ant tuberculosis treatment [2].

### Participants

Source populations were all newly diagnosed HIV positive individuals between December 2018 and July 30, 2019, in Gojjam, Northwest Ethiopia. The study population were all adult HIV positive individuals from the selected health facilities in Gojjam, northwest Ethiopia and those who fulfilled the inclusion criteria. All adults who gave consent to participate in the study were included in the study.

Eligibility criteria were, all adults (> = 18 years) who were newly diagnosed as HIV positive in the selected health facilities were included in this study. The HIV test can be in the same health facility or referred from other health facilities or community settings. Patients enrolled in treatment 7 days prior to data collection were included in the data collection. While the exclusion criteria were that treatment interruption and re-start as “new” (for example as in the case of mothers in the previous PMTCT guideline before option B+), health facilities with less than five new patients per month were excluded to use the available resources effectively.

The sample size was determined using both a single population formula for the first objective (prevalence of same-day ART acceptance) and double population formula for second objective (the associated factors) then the highest sample size was considered. Prevalence of same-day ART acceptance (54.2%) was used from South African study [15]. Accordingly, the sample size using single population proportion formula was 381 study participants were required. By adding a 10% attrition rate, it was 421. For the second objective, double population proportion formula was used and assumptions like 80% power, 95% confidence interval and 5% non-response rate were considered. Proportions used in this formula were from primary and secondary clinical outcomes (P1 = 66.10% exposed/delayed ART initiation) and (P2 = 56.21non exposed/early initiation of ART) from a previous study [33] the largest sample size was 844.

### Variables of the study

The dependent variable was same-day ART initiation with categorical response of (Yes, No) while the independent variables were grouped into two. Namely individual level and community or health facility level variables. From the individual level variables, socio-demographic characteristics such as age, sex, religion, ethnicity, marital status, educational status, occupation, wealth status, living arrangement, have child/children and family size. Baseline clinical characteristics which included opportunistic infections, body mass index (BMI), functional status, pregnancy status, any current health complaints, WHO clinical stage and mode of HIV testing (VCT or PICT). Behavioral characteristics were sero-status of sexual partner, disclosure status, and HIV test history, number of sexual partner/s, condom utilization, and alcohol use. HIV and ART related Knowledge, history of STI. Psychological characteristics were identified via perceived stigma and psychological distress. In addition to this, we documented dates of HIV test confirmed, enrolment to HIV care and ART start. Community and health facility level variables were patient residence (rural, urban), zones (East Gojjam, West Gojjam and Bahir Dar City Administration), health facility type (health center or hospital), and distance from health facility, tested in the same facility and living within the catchment area.

### Data collection instruments

In this study, structured questionnaires were used to collect information during the enrolment period. Data collection tool was developed by reviewing similar literature [18, 21, 22, 41, 48–53] and from Ethiopian HIV/ART care intake form, ART/HIV care follow up form and ART cohort registrations [2]. Psychological distress tool was adopted from Kessler-10 scale [54] which has been validated in Ethiopia [43].

### Sampling technique

**Fig. 1:**
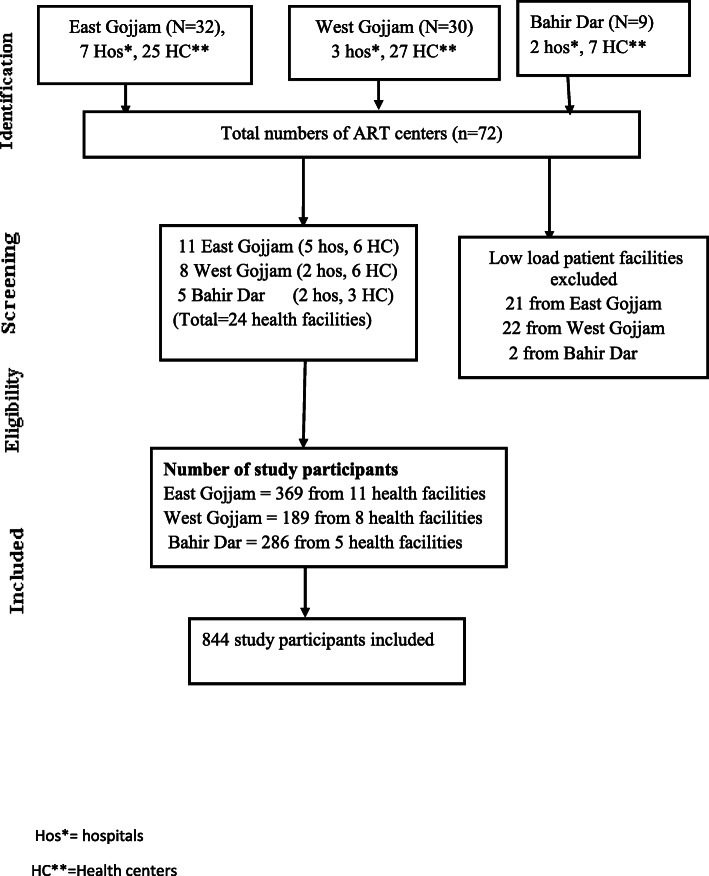
Newly diagnosed HIV positive people uptake of same-day ART in Ethiopia, 2019 Data collection procedure

In the three administrative zones, there are 72 public health facilities rendering ART service. We took 3 months of performance reports of all health facilities to estimate an adequate sample size given the available time and resources. From a total of 24 health facilities, based on their monthly average patient flow and using the previous three-month report, we estimated 8 months data collection period. We divided the total sample size over the health facilities proportionate to the patient flow. A consecutive sampling technique was used to access study participants. Hence we included 24 health facilities with an average case flow of three to five new cases per month (11 from East Gojjam, 8 from west Gojjam and 5 from Bahir Dar (see Fig. 1).

Data were collected on the same-day of HIV diagnosis or in the next visit (within 7 days). Research assistants were nurses working in the ART clinic. A consecutive sampling technique was used to access the study participants. Patient exit interview was conducted in a quiet separate room.

### Data quality assurance

Research assistants were nurses who have received training on the Ethiopian HIV/ART guideline and working in the ART clinic. Two days of training was given about the objectives of the research, methods of data collection, patient right, informed consent, confidentiality and client approach. To make the training concept uniform and to be cost-effective all recruited research assistants were invited to a central place for the training. The training also involved a field practicum in ART clinics. Pretest of the data collection tools was conducted among 5% of the sample in Wuseta Health Centre (Debre Markos) that was not included in the final data collection. Concept clarity, logical order and reliability was tested. The time required to undertake the interview was also judged and necessary adjustments were made. To minimize social desirability bias, we have employed patient exit interview in a quiet separate room and discussed the objectives of the study. However, some patients were not ready due to the “emergency” nature of learning their HIV status. In this case, we allowed the patients to absorb the meanings of test result for additional few days before interview.

### Data processing and analysis

Data was entered into EPI-Data version 3.5 and were exported to SPSS version 24 and STATA version 14 for further analysis. Data were cleaned and descriptive analysis carried out using SPSS version 24. Bivariate and multivariate logistic regression analysis were carried out using STATA version 14. The Hosmer and Lemeshow’s goodness-of-fit statistic was computed and a larger *p*-value (greater than 0.05) was considered for further analysis. We have also used receiver operating characteristics curves (ROC) to measure the discriminatory capacity of the final model. In the bivariate analysis, we have assessed the crude association using each independent variable with the outcome variable (same-day ART initiation) and those variables with *p*-value less than 0.25 included in the multivariate logistic regression model. Finally, a statistical association was declared using adjusted odds ratio (AOR) with 95% confidence interval (CI) at p-value less than 0.05.

### Operational definitions

The same-day initiation of ART was defined as acceptance of ART on the same-day of HIV diagnosis and return to home with prescribed medication (ART). Those who answered > 2 from the five HIV modes of transmission questions were categorized as having good knowledge and < 2 as poor knowledge [49]. Similarly, those who answered > 2 from the five modes of HIV prevention questions were considered as having good knowledge and < 2 as having poor knowledge [49]. Patients’ knowledge of ART score above the mean score was considered as knowledgeable. Patient’s perceived stigma score above the mean score was considered as high perceived stigma. Psychological distress was measured using Kessler (10); people who score under 20 are likely to be well, score 20–24 are likely to have a mild mental disorder, and score 25–29 are likely to have a moderate mental disorder and score 30 and over are likely to have a severe mental disorder [54].

### Ethical considerations

The proposal was approved by the joint University of Ibadan/University College Hospital, Ibadan Institutional Review Board (IRB) of the College of Medicine, University of Ibadan, Nigeria (reference number UI/EC/18/0463). It was also approved by IRB at Debre Markos University (reference number HSC/30/02/2011). We obtained written informed consent from each study subject.

## Results

### Socio-demographic characteristics

A total of 759 participants were included in this study with a response rate of 90%. About 436 (57.40%) were females. The mean age of study participants was 33.02 with a standard deviation (SD) of + 9.16 years. The majority 719 (94.70%) were Ethiopian Orthodox Christian followers by religion and 747 (98.4%) were Amhara by ethnicity. Four hundred ninety six (65.30%) were employed. More than half 397 (52.30%) had more than one child (Table 1).

**Table 1:** Socio-demographic characteristics of newly initiated HIV patients in northwest Ethiopia, 2019

Characteristics	Frequency (%)
**Sex**	
Male	323 (42.60)
Female	436 (57.40)
**Age in years**	
18–24	106 (14)
25–34	350 (46.10)
> =35	303 (39.90)
**Marital status**	
Single	228 (30)
Married	278 (36.60)
Divorced	202 (26.60)
Widow/d	51 (6.70)
**Religion**	
Orthodox Christian	719 (94.70)
Muslims	33 (4.30)
Protestant	7 (0.90)
**Educational status**	
No formal education	282 (37.20)
Primary education	249 (32.80)
Secondary or higher	228 (30)
**Employed status**	
Employed	496 (65.30)
Not employed	263 (34.70)
**Occupation**	
Farmer	137 (18.10)
Private employed	89 (11.70)
Merchant/business	157 (20.70)
Government employee	101 (13.30)
Daily labor	170 (22.40)
Student	24 (3.20)
House wife	69 (9.10)
Others^a^	12 (1.60)
**Have > 1 child**	
Yes	397 (52.30)
No	362 (47.70)
**Wealth quintile status**	
Poorest	168 (22.13)
Poorer	136 (17.92)
Middle	149 (19.63)
Wealthier	158 (20.82)
Wealthiest	148 (19.50)

Others^a^ = (carpenter, handicraft men)

### Health facility and community-related characteristics of study participants

About 331 (43.60%) were recruited from East Gojjam zone and 381 (50.20%) were selected from hospitals. Majority 577 (76%) were urban residents with 592 (78%) of them living within less than one-hour walking distance from the health facility. About 584 (76.90%) were tested HIV in the same facility in which they were linked to ART service while 513 (67.60%) were diagnosed following providers initiated testing and counseling (Table 2).

**Table 2:** Health facility and community related characteristics of HIV patients in Gojjam, northwest Ethiopia, 2019

Characteristics	Frequency (%)
**Zone**	
East Gojjam	331 (43.60)
West Gojjam	170 (22.40)
Bahir Dar city	258 (34.00)
**Facility type**	
Health center	378 (49.80)
Hospital	381 (50.20)
**Residence of the patient**	
Rural	182 (24)
Urban	577 (76)
**Distance form health facility**	
< 1 h walking	592 (78)
> =1 h walking	167 (22)
**HIV diagnosis made**	
The same facility	584 (76.90)
Another facility	175 (23.10)
**Testing modality**	
Provider initiated	513 (67.60)
Self-initiated	246 (32.40)

### Baseline clinical characteristics

Three hundred eighteen (41.90%); (95% CI, 38.2 to 45.20%) were initiated on ART on the same-day of HIV diagnosis. About 550 (72.50%) had no opportunistic infections at baseline. Majority 505 (73.60%) has BMI greater than 18.5 (Table 3).

**Table 3:** Baseline clinical characteristics of HIV patients in northwest Ethiopia, 2019

Characteristics	Frequency (%)
**Same-day ART initiated**	
Yes	318 (41.90)
No	441 (58.10)
**ART initiated within seven days of Dx.**	
Yes	498 (65.60)
No	261 (34.40)
**Reason of differing same-day ART (*n*** **= 441)**	
Not ready**	383 (86.80)
OI disease	58 (13.20)
Opportunistic infection (OI**)	
Yes	209 (27.50)
No	550 (72.50)
**BMI**	
< 16	39 (5.70)
16–18.5	142 (20.70)
> 18.5	505 (73.60)
**Functional status**	
Working	659 (86.82)
Ambulatory/bedridden	100 (13.18)
**Symptomatic presentation**	
Yes	277 (36.50)
No	482 (63.50)
**WHO clinical stage**	
I/II	569 (75)
III/IV	190 (25)
**OI prophylaxis within 7 days of diagnosis**	
Cotrimoxazole only	150 (19.80)
Isoniazide preventive therapy only	285 (37.50)
CPT and INH	67 (8.80)
No prophylaxis	257 (33.91)
**Pregnancy status (*n*** **= 436)**	
Pregnant	46 (10.61)
Not pregnant	390 (89.40)

Not ready** = (reasons were: need time to discuss with family, competing health priority), OI** = opportunistic infections such as tuberculosis, diarrheal disease, fungal disease and others

### Behavioral characteristics

Majority, 572 (75.4%) of patients had disclosed their HIV status to someone. About 745 (98.2%) had a history of sexual intercourse. Four hundred twenty nine (57.6%) of the patients have sexual partners with unknown HIV status. About 548 (73.6%) had never used condoms (Table 4).

**Table 4:** Behavioral characteristics of HIV patients in Gojjam, northwest Ethiopia, 2019

Characteristics	Frequency (%)
**Disclosed HIV status**	
Yes	572 (75.4)
No	187 (24.6)
**Living arrangement**	
Living with someone	484 (63.8)
Living alone	275 (36.2)
**Ever had sexual intercourse**	
Yes	745 (98.2)
No	14 (1.8)
**Partner HIV status (*n*** **= 745)**	
HIV Positive	229 (30.7)
HIV negative	87 (11.7)
Unknown	429 (57.6)
**Life time sexual partner (*n*** **= 745)**	
One	305 (40.9)
more than one	440 (59.1)
**Sexually active in the last 3 months**	
Yes	398 (53.4)
No	347 (46.6)
**No. of sexual partners last 3 months**	
One	356 (89.4)
more than one	42 (10.6)
**Ever use condom (*n =*** **745)**	
Yes	197 (26.4)
No	548 (73.6)
**Frequency of condom use (*n*** **= 197)**	
Most of the time	39 (19.8)
Often	45 (22.8)
Sometimes	113 (57.4)
**Condom in the most recent sex (*n =*** **197)**	
Yes	103 (52.3)
No	94 (47.7)
**Sex out of regular partner (*n =*** **745)**	
Yes	161 (21.6)
No	584 (78.4)
**Current STI (*n =*** **745)**	
Yes	49 (6.6)
No	696 (93.4)
**Ever use alcohol**	
Yes	546 (71.9)
No	213 (28.1)
**Current use of alcohol**	
Yes	172 (31.5)
No	374 (68.5)

### HIV/ART related knowledge of respondents

Patients were asked to list all possible ways of HIV transmission, prevention mechanisms and ART related issues. Six possible answers were determined for ways of HIV transmission while five items were for HIV prevention. We have calculated Cronbach alpha (α) for each knowledge related responses and found α = 0.81 for HIV transmission questions and α =0.78 for HIV prevention questions. Similarly, we have used six-item question to discriminate ART related patient knowledge and find α = 0.76. Cut of points were considered at the mean value of each score of respondent knowledge questions. From a total of the respondent, 477 (62.85%), 516 (67.98%) and 478 (62.8%) have good knowledge about ways of HIV transmission, HIV prevention and ART related knowledge respectively.

### Level of psychological distress and perceived stigma

Patients’ initial level of mental distress was found to be well (371, 41.77%), mildly distressed (173, 22.79%), moderately distressed (136, 17.92%) and severely distressed (133, 17.53%). The reliability of the tool was (α = 0.90). Generally, 371 (41.77%) of patients were well and 442 (58.23%) were mentally distressed. Similarly, level of perceived HIV related stigma was measured using the tool with a reliability score of (α = 0.61) and 599 (78.92%) were found to have a high level of perceived stigma while 160 (21.08%) had a low level of perceived stigma.

### Goodness of fit of the model

We used the receiver operating characteristics curve (ROC) to measure goodness of fit of the model. The area under ROC curve was 76.49%.

### Factors associated with uptake of same-day ART initiation

In the bivariate logistic regression, the role of several independent variables associated with the outcome variable at *p*-value less than 25% were analyzed. These included factors from the socio-demographic variables (educational status, occupation and wealth index), factors from the health facility and community-related factors (zone, types of health facility, patient residence, where HIV was diagnosed and HIV test modality) and behavioral related variables (which included having multiple sexual partners, sexually active in the last 3 months, ever use of condom, had sex out of regular sexual partner and ever use of alcohol). Other independent variables included knowledge of HIV prevention methods and level of perceived stigma and, finally clinical related variables (baseline WHO clinical stage, pregnancy status and baseline opportunistic diseases) (Table 5).

**Table 5:** Factors associated with uptake of same-day ART initiation in northwest Ethiopia, 2019

Characteristics	Same-day initiated	Not initiated	COR 95% CI	AOR (95% CI)	***P***-value
**Educational status**					
No formal education	126	156	1	1	
Primary education	121	128	1.17 (0.83–1.65)	0.99 (0.56–1.74)	0.987
Secondary or higher	71	157	**0.56 (0.39–0.81)**	0.86 (0.46–1.62)	0.650
**Occupation (*n =*** **496)**					
Farmer	65	72	1	1	
Private employed	34	55	0.68 (0.39–1.18)	1.91 (0.68–5.32)	0.215
Government	34	67	**0.56 (0.33–0.96)**	1.14 (0.39–3.31)	0.805
Merchant/business	72	85	0.94 (0.59–1.48)	1.67 (0.70–3.95)	0.242
Daily labor	79	91	0.96 (0.61–1.51)	1.21 (0.53–2.77)	0.643
Student	8	16	0.55 (0.22–1.38)	0.94 (0.17–5.13)	0.945
House wife	18	51	**0.39 (0.21–0.74)**	0.59 (0.23–1.52)	0.279
Others*	8	4	2.22 (0.64–7.70)	2.54 (0.31–20.99)	0.388
**Wealth quintile status**					
Poorest	82	86	1	1	
Poorer	61	75	0.85 (0.54–1.34)	0.69 (0.34–1.37)	0.290
Middle	58	91	0.67 (0.42–1.04)	0.62 (0.29–1.28)	0.195
Wealthier	59	99	**0.63 (0.40–0.97)**	0.94 (0.44–2.02)	0.874
Wealthiest	58	90	0.68 (043–1.05)	0.67 (0.30–1.49)	0.330
**Zone**					
East Gojjam	129	202	1.13 (0.81–1.59)	0.97 (0.55–1.72)	0.928
West Gojjam	96	74	**2.30 (1.55–3.42)**	**2.04 (1.04–3.97)**	**0.037**
Bahir Dar city	93	165	**1**	1	
**Facility type**					
Health center	222	156	**4.22 (3.10–5.75**)	**3.06 (1.90–4.92)**	**< 0.001**
Hospital	96	285	1	1	
**Residence of the patient**					
Rural	89	93	**1.45 (1.04–2.03)**	1.84 (0.94–3.60)	0.075
Urban	229	348	**1**	0	
**HIV was diagnosed at**					
The same facility	260	324	**1.62 (1.14–2.31)**	**2.16 (1.24–3.74)**	**0.006**
Another facility	58	117	**1**	**1**	
**Testing modality**					
Provider initiated	199	314	**1**	1	0.782
Self-initiated	119	127	**1.48 (1.09–2.01)**	0.93 (0.56–1.52)	
**Life time sexual partner (*n =*** **745)**					
One	141	164	1.31 (0.98–1.76)	1..41 (0.87–2.27)	0.162
more than one	174	266	1	1	
**Sexually active 3 months**					
Yes	184	214	**1.42 (1.06–1.90)**	1.30 (0.80–2.13)	0.279
No	131	216	**1**	1	
**Ever use condom (*n =*** **745)**					
Yes	65	132	**1**	1	
No	250	298	**1.70 (1.21–2.40)**	1.61 (0.87–3.00)	0.131
**Sex out of regular partner (*n =*** **745)**					
Yes	59	102	**1**	1	
No	256	325	**1.35 (0.94–1.93)**	0.76 (0.39–1.49)	0.425
**Ever use alcohol**					
Yes	215	331	**1**	1	
No	103	110	**1.44 (1.04–1.98)**	1.45 (0.90–2.34)	0.126
**Opportunistic infection**					
Yes	65	144	**1**	**1**	
No	253	297	**1.89 (1.35–2.64)**	**2.08 (1.04–4.19)**	**0.039**
**WHO clinical stage**					
I/II	264	305	**2.18 (1.53–3.11)**	0.80 (0.38–1.64)	0.542
III/IV	54	136	**1**	1	
**Pregnancy status (*n =*** **436)**					
Pregnant	31	15	**3.20 (1.67–6.13)**	**3.97 (1.78–8.87)**	**0.001**
Not pregnant	153	237	**1**	**1**	
**Living arrangement**					
Living with someone	211	273	**1.21 (0.89–1.64)**	1.36 (0.83–2.21)	0.220
Living alone	107	168	**1**		
**HIV prevention knowledge**					
Good	198	318	**1**	1	
Poor	120	123	**1.57 (1.15–2.13)**	1.10 (0.67–1.79)	0.696
**Perceived stigma**					
High	262	337	**1.44 (1.01–2.07)**	1.23 (0.68–2.22)	0.488
Low	56	104	**1**	1	

In the multivariate logistic regression, about five variables were statistically significant. Patients in West Gojjam Zone were 2.04 times more likely to initiate ART compared to those in Bahir Dar city administration [AOR, 2.04, (95% CI, 1.04–3.97)]. Similarly, patients in the health center were 3.06 times more likely to accept same-day ART initiation compared to those in the hospital [AOR, 3.06, (95% CI, 1.90–4.92)]. Patients who had their HIV status diagnosed the same health facility where they were linked for ART were 2.16 times more likely to initiate the same-day of diagnosis. Finally, patients with no opportunistic disease and pregnant women were more likely to initiate ART (Table 5).

## Discussion

This study found that the magnitude of same-day ART initiation was 41.9% (95% CI, 38.2–45.20%) and factors associated with same-day ART initiation were having opportunistic infections, being pregnant, having HIV status diagnosed at the same health facility where one is linked to ART service, accessing care at a health center compared to a hospital and being in West Gojjam administrative zone.

Same-day treatment initiation was low (41.9%) in the study area. But there was increasing in the proportion of newly diagnosed HIV positive people who initiated ART within 7 days of HIV diagnosis to 65.6%. The finding was in line with a study in South Africa with overall same-day ART initiation of 40.1% and initiated within 7 days to 62.86% [15] and similar with study from Taiwan reported 68% rapid ART initiation within 7 days of HIV confirmed diagnosis in 2017 [8]. However, same-day ART initiation was lower compared to the recent finding of 54.2% in June 2018 in South Africa [15], Zimbabwe (65%) [55], 94.9% in US [56] and San Francisco (96%) [57]. The high discrepancy observed between the findings of the current study and the previous studies may be due to the patients receiving multidisciplinary evaluation, support, and insurance enrollment/optimization in the San Francisco and US studies. This was not the case in our current study. Additionally, the present study settings included both primary and secondary healthcare facilities, while the study in South Africa was only from primary healthcare facilities. It must be noted that patients who present to primary healthcare facilities are more likely to have uncomplicated presentation.

On the other hand, the magnitude of same-day ART initiation and rapid ART initiation within a week were better than the previous study in San Diego which was 26 and 48% respectively [23]. The possible reason for the difference observed may be because the former study was undertaken before the WHO official recommendation of immediate ART initiation. Moreover, in a cluster-randomized trial study in rural South Africa it was found that only 36% initiated ART within a month. This implies that there is an increasing rate of rapid ART initiation after the official declaration of rapid ART following HIV confirmed test by WHO.

Patients without opportunistic infections were two times more likely to initiate same-day ART compared with patients with one or more opportunistic infections. The finding is similar to a study in South Africa which reported that patients who presented with less advanced clinical disease were more likely to accept same-day ART initiation [15]. However, such findings are in contrast with several qualitative studies that had reported the absence of symptoms or signs of ill health, which is the major reason for differing same-day ART [34–36, 58]. Moreover, Ethiopian Ministry of Health and WHO guidelines recommended that a rapid approach to ART initiation is particularly relevant to people with advanced HIV disease [1, 2] yet CD4 count determination was not available in the study area during the study period. The discrepancy may a result of healthcare providers who give priority to treat acute opportunistic infection due to fear of pill burden rather than initiating ART. This approach causes delay in treatment initiation [1]. And explains why patients who presented with TB as an opportunistic infection were mostly not initiated on same-day ART treatment (*P* < 0.002). The limitation of the current study is in addition to lack of baseline CD4 determination, it included only the same-day ART initiation and did not follow how long they delayed and did not study the healthcare providers’ behavior that affects timely ART initiation. Hence, it is better to determine the baseline CD4 count to ascertain the advancement of disease. Follow-up of those patients who differed rapid ART initiation for a reasonable time is necessary to document barriers of treatment initiation in the course of time.

Uptake of same-day ART among pregnant women was 67.4% a value lower that the report from a study in Zimbabwe in which over 80% of those who underwent HIV testing in maternal and child health departments initiated ART on the same-day [55]. Similarly, two different studies in Cape Town South Africa reported that 73% of pregnant women initiated ART same-day [30] and 91% of women initiated onto ART starting the same day treatment eligibility was determined [27]. However, the present finding was better than a study in Malawi (51 and 63%) [31] depending on service integration with HIV clinic and ANC. The difference can be attributed due to differences in socio-economic status of the patients, the study design and the study period. Most importantly, pregnant women were four times more likely to initiate same-day ART compared to the general adult HIV positive population. The reason for better acceptance of same-day ART initiation among pregnant women can be explained by the emphasis given by both WHO and the Ethiopian government to initiate immediate ART for pregnant and lactating women [1, 2]. Moreover, pregnant and lactating women are ready to accept same-day ART due to their commitment to their child’s health [25].

Newly diagnosed HIV positive patients in a health facility in which they were linked to ART service were two times more likely to initiate same-day ART compared with those tested HIV positive from another health facility and subsequently referred. This was due to the time needed to transfer from the original HIV testing site to the actual health facility rendering ART service. A significant number of our study participants knew their HIV status in private and non-governmental health facilities. Similarly, in Tanzania, HIV testing in the community setting was a reason for delayed treatment initiation [24]. This emphasizes the need for active referral mechanism between HIV testing sites and ART clinics to shorten the time between HIV diagnosis and ART initiation. Well established referral systems can increase uptake of ART [57].

New HIV patients who sought HIV treatment and support services at health centers were three times more likely to initiate same-day ART compared to those at hospitals. This finding is supported by previous studies in Ethiopia that concluded, HIV treatment in health centers was feasible and as effective as in hospitals [59–63]. Additionally, hospitals are overloaded with many patients that may cause lack of time for adequate patient preparation for ART initiation within the same-day of HIV diagnosis. Furthermore, patients treated in West Gojjam were two times more likely to initiate ART compared to the regional state capital-Bahir Dar city administration. The possible reason can be that health facilities in cities like Bahir Dar are overloaded. In addition to busy clinics, urban residents and educated people were less likely to accept ART on the days of HIV diagnosis. The implications of the above findings is in addition to patient and clinician factors affecting same-day treatment initiation, the health system management at the zonal level may also affect the program’s effectiveness at large.

### Limitations of the study

Social desirability bias may influence this study due to persistent natures of HIV related stigma in the community. Inclusion of TB patients in this study may underestimated the magnitude of SDI though there were few patients who initiated same-day ART. Some patients were not stable at the time of their HIV diagnosis and we postponed data collection to the next visit within a week. As a result, some of them did not come back to the clinic and this may have introduced bias by avoiding most at-risk patients for delayed ART initiation. As a result, the finding may not be generalized for such patients. Additionally, this study investigated factors only from the patient side characteristics and may not explain the healthcare providers’ perspective on the same-day ART initiation. Therefore, further study is recommended to uncover the role of healthcare workers in same-day ART initiation.

## Conclusion

Less than half of newly diagnosed HIV positive people initiated ART in the same-day of HIV diagnosis in Ethiopia. None of the socio-demographic, behavioral and psychological factors were associated with differing of same-day ART initiation. Rather, clinical factors such as having opportunistic infections and non-pregnancy status affected the immediate initiation of treatment. Others like being in a hospital and major city-Bahir Dar were also factors. This indicated that same-day ART initiation was affected by healthcare providers’ decision and organizational factors like patient load and the strength of health system management.

## Data Availability

The datasets used and/or analyzed during the current study are available from the corresponding author on reasonable request.

## Abbreviations

HAART: Highly Active Antiretroviral Therapy
HIV: Human immunodeficiency virus
PLHIV: People Ling with HIV
WHO: World Health Organization
